# Impact of Nano-Scale Distribution of Atoms on Electronic and Magnetic Properties of Phases in Fe-Al Nanocomposites: An Ab Initio Study

**DOI:** 10.3390/nano8121059

**Published:** 2018-12-16

**Authors:** Ivana Miháliková, Martin Friák, Yvonna Jirásková, David Holec, Nikola Koutná, Mojmír Šob

**Affiliations:** 1 Institute of Physics of Materials, Academy of Sciences of the Czech Republic, Žižkova 22, CZ-616 62 Brno, Czech Republic; ivanamihalik2@gmail.com (I.M.); jirasko@ipm.cz (Y.J.); mojmir@ipm.cz (M.Š.); 2 Department of Condensed Matter Physics, Faculty of Science, Masaryk University, Kotlářská 2, CZ-611 37 Brno, Czech Republic; nikakoutna@gmail.com; 3 Department of Materials Science, Montanuniversität Leoben, Franz-Josef-Strasse 18, A-8700 Leoben, Austria; david.holec@unileoben.ac.at; 4 Institute of Materials Science and Technology, TU Wien, Getreidemarkt 9, A-1060 Vienna, Austria; 5 Department of Chemistry, Faculty of Science, Masaryk University, Kotlářská 2, CZ-611 37 Brno, Czech Republic; 6 Central European Institute of Technology, CEITEC MU, Masaryk University, Kamenice 5, CZ-625 00 Brno, Czech Republic

**Keywords:** Fe_3_Al, Fe-Al, magnetism, interfaces, ab initio, stability, disorder

## Abstract

Quantum-mechanical calculations are applied to examine magnetic and electronic properties of phases appearing in binary Fe-Al-based nanocomposites. The calculations are carried out using the Vienna Ab-initio Simulation Package which implements density functional theory and generalized gradient approximation. The focus is on a disordered solid solution with 18.75 at. % Al in body-centered-cubic ferromagnetic iron, so-called α-phase, and an ordered intermetallic compound Fe${}_{3}$Al with the D0${}_{3}$ structure. In order to reveal the impact of the actual atomic distribution in the disordered Fe-Al α-phase three different special quasi-random structures with or without the 1st and/or 2nd nearest-neighbor Al-Al pairs are used. According to our calculations, energy decreases when eliminating the 1st and 2nd nearest neighbor Al-Al pairs. On the other hand, the local magnetic moments of the Fe atoms decrease with Al concentration in the 1st coordination sphere and increase if the concentration of Al atoms increases in the 2nd one. Furthermore, when simulating Fe-Al/Fe${}_{3}$Al nanocomposites (superlattices), changes of local magnetic moments of the Fe atoms up to 0.5 $\mu_{B}$ are predicted. These changes very sensitively depend on both the distribution of atoms and the crystallographic orientation of the interfaces.

## 1. Introduction

Fe-Al-based materials represent one of the most promising classes of alloys intended for high-temperature applications. Remarkable are, in particular, their (i) resistance to oxidation, (ii) relatively low density, (iii) electrical resistivity and (iv) low cost of raw materials [1,2,3]. Their wider use is currently hindered by their lower ductility at ambient temperatures and a drop of the strength at elevated temperatures [3]. Regarding the former issue, the brittleness has been shown to be caused by an extrinsic effect, in particular hydrogen atoms [4,5] and there are experiments showing that the Fe${}_{3}$Al could have reasonable ductility if the environmental embrittlement is eliminated [6,7].

In order to further fine-tune properties of iron-aluminium materials, they are intensively studied both experimentally and theoretically (see, for example, Refs. [8,9,10] or an excellent review of these activities published by Sundman and co-workers [11]). As far as basic thermodynamic properties are concerned, the Fe-Al phase diagram was originally determined by Kattner and Burton [12]. It reflects many phase transitions in the Fe-Al system from a disordered solid solution, A2 phase, through partially ordered, B2 phase, or ordered D0${}_{3}$ phase. This offers some possibilities to produce two-phase Fe-Al alloy, e.g., ordered D0${}_{3}$ phase embedded in B2 or disordered phase, the (nano)composite, exhibiting potentially new physical properties. From a viewpoint of applications, the final states of Fe-Al-based materials are highly sensitive to various factors including thermo-mechanical history (see, e.g., Ref. [13]). Again, it is because of many phase transitions in the Fe-Al system.

A particular sub-class of Fe-Al-based materials is represented by composites consisting of an ordered Fe${}_{3}$Al phase with the D0${}_{3}$ structure and a disordered Fe-Al solid solution with about 18–19 at.% Al. An experimental evidence of the co-existence of Fe${}_{3}$Al and a disordered Fe-Al phase has been provided, for example, by transmission electron microscopy (TEM) technique which is sensitive to anti-phase boundaries (APBs). The APBs have a different character in Fe${}_{3}$Al intermetalics and in the Fe-Al phase [14,15,16,17]. Specifically in the case of Fe-Al, the combined experimental study supported by theoretical simulations identified round/oval droplets of the disordered Fe-Al phase formed at the expense of diminishing amount of ordered Fe${}_{3}$Al phase.

Next to the experimental research reported in the above mentioned papers, various Fe-Al-based materials were theoretically studied by quantum-mechanical calculations in the last three decades (see, e.g., Refs [18,19,20,21,22,23,24,25,26,27,28,29,30,31,32,33]). A surprising controversy has appeared in the case of first-principles prediction of the ground-state structure of Fe${}_{3}$Al. For example, Lechermann et al. [34] determined the energies of Fe${}_{3}$Al in the case of both experimentally observed D0${}_{3}$ structure and a face-centered-cubic L1${}_{2}$ one and showed that neither local density approximation (LDA) nor Perdew-Burke-Ernzerhof (PBE) [35] parametrization of the generalized gradient approximation (GGA) can correctly reproduce the D0${}_{3}$ structure as the ground state of Fe${}_{3}$Al. Similarly, Connetable and Maugis [36] calculated properties of Fe${}_{3}$Al employing Perdew-Burke-Ernzerhof (PBE) parametrization [35] of the GGA and found out that Fe${}_{3}$Al has lower energy in the L1${}_{2}$ structure than in the experimentally observed D0${}_{3}$ structure. In a subsequent paper by Lechermann and co-workers [37], electronic correlations and magnetism in Fe${}_{3}$Al were studied employing LDA with an on-site Hubbard potential (LDA+U). The correct D0${}_{3}$ structure was obtained as the ground state of Fe${}_{3}$Al. As yet another example, Kellou et al. [38] compared the energies of Fe${}_{3}$Al in both D0${}_{3}$ and L1${}_{2}$ structures using the pseudopotential plane-wave selfconsistent field ab-initio package and ultra-soft pseudopotentials. In this case, the correct ground-state structure was reproduced, too. Being aware of these findings, we have carefully chosen a reliable computational set up which provides a correct ground-state structure of Fe${}_{3}$Al, i.e., the D0${}_{3}$ structure.

Our paper aims at providing an analysis of complex relations between distributions of atoms on one hand and local magnetic moments of Fe atoms on the other in different Fe-Al phases. The complexity of the magnetic states is, in particular, exemplified by (i) the anti-correlations between the local magnetic moments of the Fe atoms and the number of Al atoms in the 1st coordination shell and (ii) a completely opposite trend, correlations between the local magnetic moments and the number of Al atoms in the 2nd coordination sphere of these Fe atoms. As the main novelty of our paper we show that the relations between local magnetic moments on one hand and the atomic distributions on the other hand are further complicated by the existence of interfaces in Fe-Al nanocomposites.

## 2. Methods

**Figure 1:**
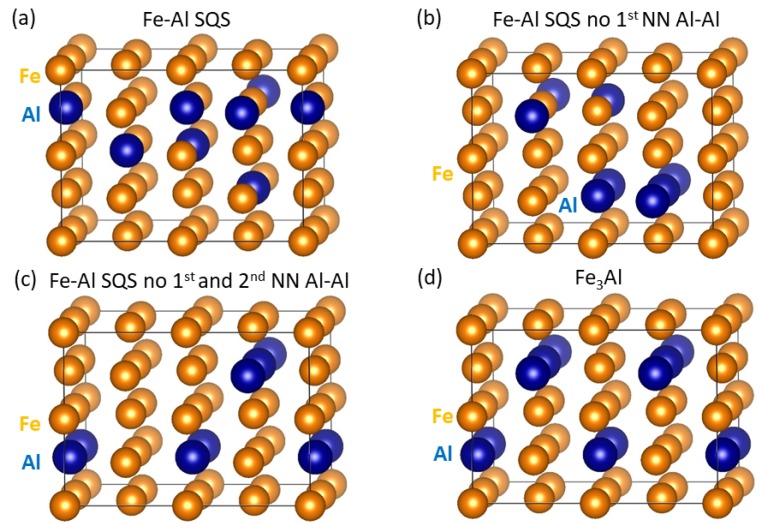
Schematic visualization of the 32-atom supercells used in our calculations: (**a**) a general special quasi-random structure (SQS) model for the Fe-Al phase (with 18.75 at.% Al), (**b**) an SQS model for the Fe-Al phase without any 1st nearest-neighbor (NN) Al-Al pairs, (**c**) an SQS model for the Fe-Al phase without the 1st and 2nd NN Al-Al pairs and (**d**) a 32-atom supercell of the Fe${}_{3}$Al intermetallics. The unrelaxed atomic positions are listed in Table A1 in the Appendix.

Our density-functional-theory [39,40] calculations employed projector augmented wave (PAW) pseudopotentials [41] and the exchange and correlation energy in the generalized gradient approximation (GGA) parametrized by Perdew and Wang [42] (PW91) with the Vosko-Wilk-Nusair correction [43] as implemented in the Vienna Ab initio Simulation Package (VASP) [44,45,46]. Plane-wave expansions were performed up to the cut-off energy of 350 eV and the Methfessel-Paxton method of the first order was adopted with a smearing width of 0.1 eV. The sampling of the Brillouin zone was done using Monkhorst-Pack [47] grids 10 × 10 × 6 and 10 × 10 × 3 for the simulation supercells containing 32 atoms (2$\sqrt{2}$× 2$\sqrt{2}$× 2 multiple of 2-atom cube-shape conventional bcc-cell) as models of individual phases (see Figure 1) and 64 atoms (double the size of 32-atom supercells—nanocomposites—see figures in Section 4). All local magnetic moments were initially oriented in a parallel manner which corresponds to the ferromagnetic state.

## 3. Results for Individual Phases

When setting up computational supercells as models for phases appearing in Fe${}_{3}$Al/Fe-Al nanocomposites, the disordered Fe-Al phase deserves a special attention. Our choice was motivated by our previous calculations of interactions of Al atoms in a bcc Fe ferromagnetic matrix [48] as well as by other theoretical results [49]. Regarding the latter, Amara and coworkers [49] studied the electronic structure and energetics of the dissolution of aluminum in α-iron and the interaction between Al atoms and vacancies. The stability of complexes containing Al and vacancy was found to be driven by strong Al-vacancy attractions and an Al-Al repulsion. Our calculations [48] also showed clear ordering tendencies of Al atoms. In particular, the energy of the system was decreasing with the elimination of the 1st and 2nd nearest neighbour (NN) Al-Al pairs, i.e., energies of the system containing the 1st and 2nd NN Al-Al pairs were higher than energies of the systems without them. Therefore, in this study, we compare properties obtained from three models which differ in the number of Al-Al pairs and we employed the concept of special quasi-random structure (SQS) [50] generated in USPEX code [51,52,53]. First, a general SQS (Figure 1a) containing the 1st and 2nd NN Al-Al pairs (A2-like with respect to Al-Al pairs) is used. Second, an SQS without any 1st NN Al-Al pairs (Figure 1b, B2-like w.r.t. Al-Al pairs) is utilized. Finally, an SQS without the 1st and the 2nd NN Al-Al pairs (effectively an Fe-rich Fe${}_{3}$Al) (Figure 1c, i.e., D0${}_{3}$-like w.r.t. the Al-Al pairs) is studied. We used 32-atom supercells which allow for a wider range of distribution of aluminium atoms in the disordered Fe-Al phase and the three different models have the stoichiometry Fe${}_{26}$Al${}_{6}$ (Figure 1a–c). The ordered intermetallic compound Fe${}_{3}$Al is modeled by a 32-atom supercell with the stoichiometry Fe${}_{24}$Al${}_{8}$ (Figure 1d).

The thermodynamic stability of the Fe-Al polymorphs was assessed from their computed energies *E* by evaluating the formation energy: $E_{f}$(Fe${}_{x}$Al${}_{y}$) = (*E*(Fe${}_{x}$Al${}_{y}$) −x·E(Fe)−y·E(Al))/(*x* +*y*) where *x* and *y* are numbers of Fe and Al atoms in the supercells and E(Fe), E(Al) are their chemical potentials, i.e., energies of elemental ferromagnetic (FM) body-centered cubic (bcc) Fe and non-magnetic (NM) face-centered cubic (fcc) Al. The computed formation energies, which partly appeared in Ref. [48], are −0.119 eV/atom (Figure 1a, A2-like), −0.121 eV/atom (Figure 1b, B2-like) and −0.144 eV/atom (Figure 1c, D0${}_{3}$-like). The supercell with the least disordered distribution (Figure 1c) and Al atoms further apart is thus thermodynamically the most stable. This finding is in agreement with the Al-Al repulsion discussed in Refs. [48,49]. The other two (A2-like and B2-like) atomic distributions can be possibly considered as models for high-temperature states.

**Figure 2:**
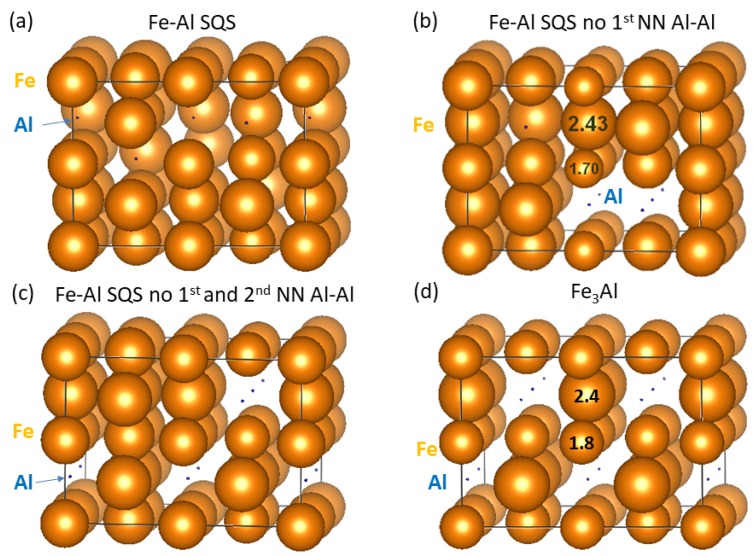
Schematic visualization of local atomic magnetic moments calculated for individual phases (see Figure 1): (**a**–**c**) are SQS models for the Fe-Al phase with different atomic arrangements and (**d**) for the Fe${}_{3}$Al. The moments are visualized so that the diameters of the spheres reflect the magnitudes of the local magnetic moments (a few examples are in parts (**b**,**d**) in $\mu_{B}$). Magnetic moments of Al atoms are so small, less than 0.05 $\mu_{B}$, that they are shown only as blue dots.

As one of the prime topics of our current study we examine relations between the value of local magnetic moments of Fe atoms and their surroundings. Figure 2 displays the spatial distribution of magnetic moments in the four studied phases with the diameter of spheres representing the magnitude of local magnetic moments. It is rather difficult to extract a clear pattern in the case of the three Fe-Al SQS polymorphs (Figure 2a–c) similarly as in our study of Fe-Al with different Al concentrations [20]. However, two different Fe sublattices in Fe${}_{3}$Al with different values of local magnetic moment are easily recognizable in Figure 2d. One sublattice contains Fe atoms surrounded by 4 Al and 4 Fe atoms while the other has the Fe atoms surrounded by 8 Fe atoms. The local magnetic moments of the Fe atoms on the former sublattice are smaller (1.8 $\mu_{B}$) than those corresponding to the latter case (2.4 $\mu_{B}$).

**Figure 3:**
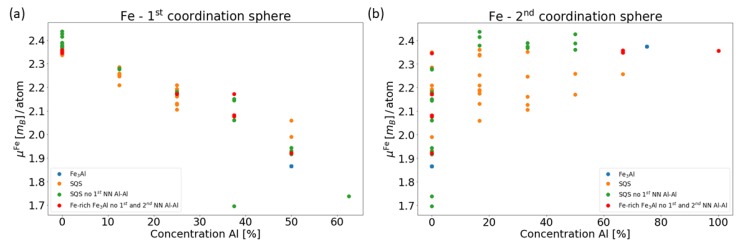
Dependences of local magnetic moments of Fe atoms in all studied phases (shown in Figure 1) as functions of concentration of Al atoms in the 1st (**a**) and the 2nd (**b**) coordination shell, respectively.

In order to provide an overall description of all Fe atoms shown in Figure 1 and their corresponding local magnetic moments in Figure 2, we show them as functions of the concentration of Al atoms in Figure 3. In particular, the moments are found to decrease with increasing concentration of Al atoms in the 1st coordination shell, i.e., the fraction of 8 atoms in total in this shell, see Figure 3a.

A similar finding, decreasing local magnetic moments with increasing number of Al atoms in the 1st nearest neighbor (NN) shell was also reported across a wider range of Al concentrations in Ref. [20]. Very interestingly, an opposite trend, i.e., an increase of the local magnetic moments of the Fe atoms as a function of the concentration of Al atoms (fraction out of 6 atoms in total), is obtained in the case of 2nd NN shell, see Figure 3b. These results clearly show a multi-faceted sensitivity of local magnetic moments of Fe atoms to the distribution of atoms in their local surroundings.

**Figure 4:**
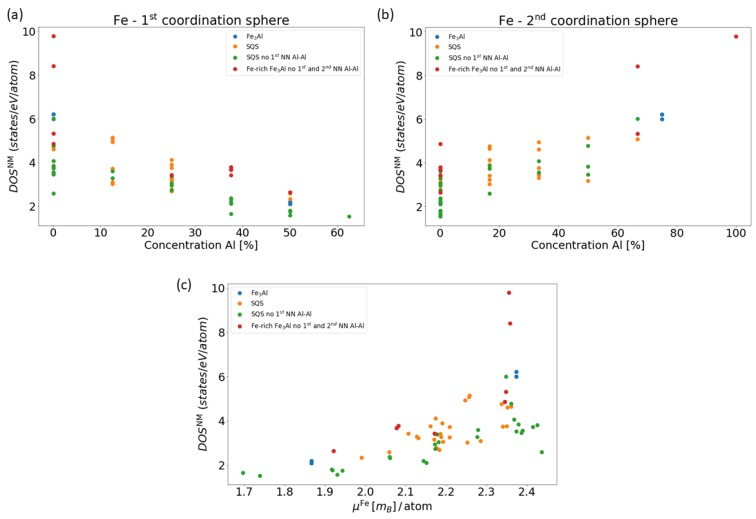
Calculated dependences of the density of states (DOS) of the Fe atoms at the Fermi level $E_{F}$ in the case of NM states as a function of the Al concentration in the 1st (**a**) and the 2nd (**b**) coordination shell, respectively. Part (**c**) shows the DOS of the Fe atoms at the $E_{F}$ in the case of NM states as a function of their local magnetic moments in the case of FM states.

In order to shed light on the opposite trends of local magnetic moments of the Fe atoms as a function of the Al concentration in the 1st and 2nd coordination sphere, we recall the Stoner model which connects the value of the density of states at the Fermi level in a non-magnetic state with the tendency to spin polarization. We have treated all four studied phases (see Figure 1) as non-magnetic and determined the local DOS of individual Fe atoms at the Fermi level in the case of non-magnetic cases. The application of the Stoner model to individual atoms (see, e.g., a previous study [54]) is possible here as (i) contributions of Al atoms to the DOS at the Fermi level are small and (ii) the Stoner model is related to *d*-states rather than to *s*-/*p*-states. Our results are shown in Figure 4.

The values of DOS depicted in Figure 4 as functions of the Al concentration in the 1st or 2nd NN shell are similar to those shown in Figure 3. The local densities of states at the Fermi level of non-magnetic Fe atoms indeed resemble the trends of the local magnetic moments of these Fe atoms when they are spin-polarized, see Figure 4c.

**Figure 5:**
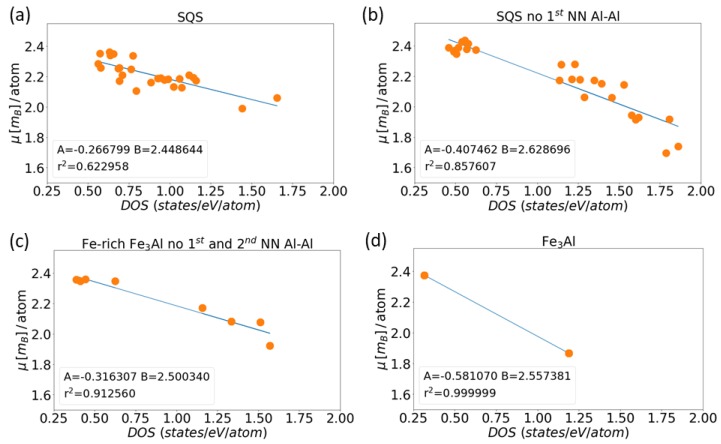
The dependences of local magnetic moments of Fe atoms on the density of states of these atoms (A and B represent coefficients in a linear fit y=A × x+B and $r^{2}$ is the coefficient of determination): (**a**) a general Fe-Al SQS, (**b**) an SQS without the first NN Al-Al pairs, (**c**) Fe-rich Fe${}_{3}$Al without the first and the second nearest neighbour Al-Al pairs, and (**d**) Fe${}_{3}$Al.

As a next step we analyze local magnetic moments of Fe atoms as a function of their local atomic density of states at the Fermi level in the case of magnetic calculations (we consider the DOS as the sum of both spin channels). In contrast to the Stoner-like model elaborated above when we examined DOS of individual atoms in the non-magnetic state we below analyze the local DOSes of individual atoms in their spin-polarized, i.e., magnetic, states. The computed data points are shown in Figure 5. Both quantities are clearly anti-correlated. As they roughly follow linear trends, we use the least-square method to find suitable linear fitting functions. Interestingly, the slopes of these linear fitting functions, which are called parameter *A* in Figure 5, are significantly different for the three SQS Fe-Al variants (Figure 5a–c) on one hand and the ordered phase Fe${}_{3}$Al (Figure 5d) on the other hand. There are differences in the slope among the three Fe-Al SQS variants, too, but differences within this group of three values (*A* = −0.27, −0.32 and −0.41 $\mu_{B}$/state/eV) are relatively smaller than the difference between this group and the slope in the case of the Fe${}_{3}$Al (*A* = −0.58). The difference between (i) the group of slope values related to the three Fe-Al SQS variants and (ii) the slope related to Fe${}_{3}$Al can be tentatively attributed to the difference in the Al concentration (18.75 vs. 25 at.%). But the difference of the slopes of the three Fe-Al SQS variants is most likely a consequence of different distribution of atoms because the concentration of Al is equal in all three of them.

Besides, the local magnetic moments of Fe atoms differ significantly within the phases in question. This sensitivity of these moments is easily recognizable in the case of the ordered Fe${}_{3}$Al phase where the moments are equal either to 1.8 or 2.4 $\mu_{B}$. These different values stem from the existence of two different crystallographic sublattices of Fe atoms in the ordered Fe${}_{3}$Al compound. Consequently, these sublattices represent qualitatively different chemical environments (4 Al + 4 Fe vs. 8 Fe atoms in the 1st coordination shell as discussed above). For the disordered Fe-Al SQS polymorphs the local magnetic moments cover wider ranges of values. For the general SQS the values of magnetic moments are between 1.9–2.4 $\mu_{B}$, for the SQS without the 1st NN Al-Al pairs it is the widest obtained range from 1.6 to 2.45 $\mu_{B}$ and for the Fe-rich Fe${}_{3}$Al without the 1st and the 2nd nearest neighbor Al-Al pairs the range is 1.8–2.4 $\mu_{B}$.

**Figure 6:**
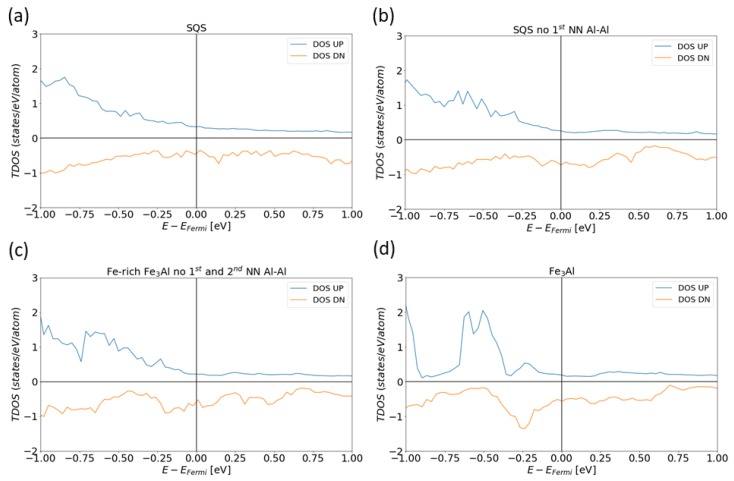
Calculated total densities of states (TDOS) in the case of the 32-atom supercells as models for individual phases: figures (**a**–**c**) are SQS models for the Fe-Al phase with different atomic arrangements and figure (**d**) for the Fe${}_{3}$Al intermetallic compound.

The densities of states not only at the Fermi level but also at other energies are shown in Figure 6. The studied systems contain electrons with two opposite spin orientations which we further on refer to as UP and DOWN channels and analyze the DOS for each of them separately. In the case of spin UP-channel electrons of the four studied systems, the total DOSs (TDOSs) are qualitatively rather similar and do not exhibit any particularly noticeable features. In contrast, the spin DOWN-channel DOS exhibits specific trends. It appears that for the general SQS (Figure 6a) and the SQS without the 1st NN Al-Al pairs (Figure 6b) the densities of states have notable local maxima at the Fermi level when compared with that of the SQS without any 1st and 2nd NN Al-Al pairs (Figure 6c). This can be possibly linked to a higher thermodynamic stability (lower energy) of the SQS without the 1st and 2nd NN Al-Al pairs which was found in our previous study [48]. Note that as the DOWN-spin DOSs are depicted as negative values, the maxima appear as local minima.

After studying individual Fe-Al and Fe${}_{3}$Al phases separately, we next examine changes induced in the local magnetic moments of the Fe atoms when forming their nanocomposites.

## 4. Results for Nanocomposites

**Figure 7:**
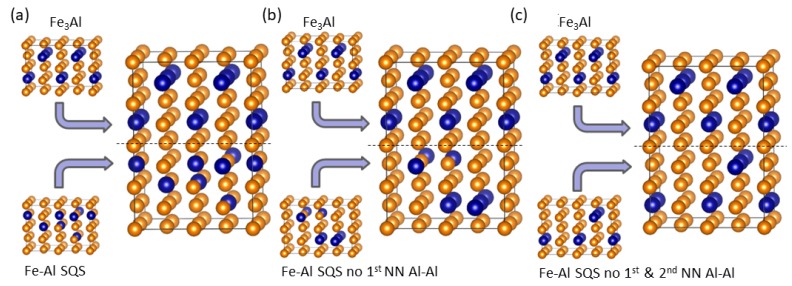
Visualization of nanocomposite supercells for the (001) interface plane. The figures show how the Fe${}_{3}$Al intermetallic compound is combined with different models of the Fe-Al phase with 18.75 at.% Al, in particular the general SQS (A2-like) model (**a**), the SQS model without the 1st NN Al-Al pairs, B2-like, (**b**) and the SQS without the 1st and 2nd NN Al-Al pairs, D0${}_{3}$-like (**c**).

**Figure 8:**
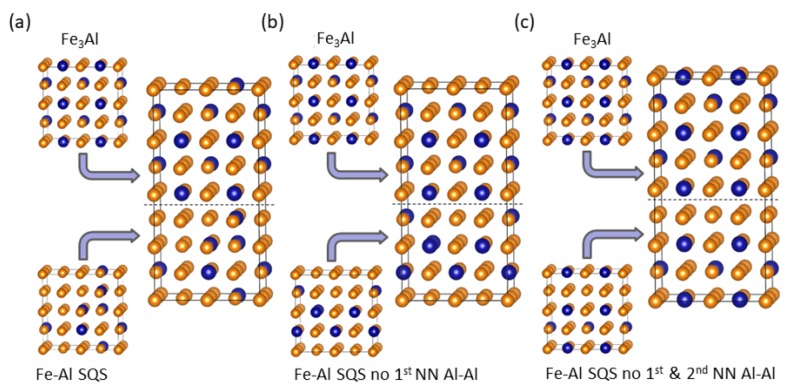
The same as in Figure 7 but for nanocomposite supercells with the (110) interface plane.

The supercells of individual phases shown in Figure 1 have the facets with (001), (110) and (1$\bar{1}$0) crystallographic orientations (with respect to a two-atom conventional cubic cell of bcc Fe). Therefore, when combining three different polymorphs of Fe-Al with the Fe${}_{3}$Al intermetallic compound three different nanocomposites with different interfaces between the phases can be simulated. They are schematically visualized in Figure 7 and Figure 8 (the construction in the case of the (1$\bar{1}$0) interface plane is not shown as it is rather similar to that of the (110) one). It is worth noting that due to the periodic boundary conditions applied in our calculations, the simulated nanocomposites form so-called superlattices [55,56,57,58,59,60,61,62,63,64,65,66,67,68,69,70,71,72,73,74,75,76] when both phases coherently co-exist and the atomic planes continue from one phase into another. As another consequence of (i) the periodicity and (ii) the fact that the supercells modeling the Fe-Al phase are disordered, the two interfaces per 64-atom supercell are not the same. The scalar properties (e.g., the interface energy γ below) are then averages of the two interfaces.

Regarding the thermodynamic properties of the studied interfaces, they were assessed in Ref. [48]. In particular, the interface energies γ (in fact, their averages—see the discussion above) were evaluated according to the formula γ = (*E*(Fe${}_{3}$Al/Fe-Al) −*E*(Fe${}_{3}$Al) −*E*(Fe-Al))/(2S) from the energy of the composite system *E*(Fe${}_{3}$Al/Fe-Al), the energies of individual phases, *E*(Fe${}_{3}$Al) and *E*(Fe-Al) and the area of the interfaces *S*. The interface energies turned out to be very weakly dependent on the crystallographic orientation of interfaces (see Ref. [48]). For example, for the nanocomposites containing the general SQS, see Figure 1a, the interface energies are equal to 0.019 J/m${}^{2}$, 0.020 J/m${}^{2}$ and 0.022 J/m${}^{2}$ for the (001), (110) and (1$\bar{1}$0) orientation, respectively. It should be noted that these very low interface energies are quite close to an estimated error-bar of our calculations, about 0.005 J/m${}^{2}$.

The fact that the interface energies are so similar for different orientations is in line with the findings of Oguma et al. [17] who identified round/oval droplets of the disordered Fe-Al phase surrounded by the Fe${}_{3}$Al phase. The rounded shape of these droplets can be probably connected with the fact that interface energy is not sensitive to crystallographic orientation and, therefore, the interfaces studied in this paper are equally probable as others. It should be noted that other properties, such as mechanical ones, can be much more sensitive with respect to the orientation. Further the interface energies sensitively depend on the distribution of atoms and become practically zero (within the error-bar of our calculations, i.e., about 0.001 J/m${}^{2}$) for nanocomposites containing Fe${}_{3}$Al and the Fe-Al variant without the 1st and 2nd NN Al-Al pairs (a Fe-rich variant of the Fe${}_{3}$Al). This unusual result can be explained by the fact that Fe${}_{3}$Al can contain rather high concentration of point defects (such as off-stoichiometric Al atom, anti-sites) and covers rather broad range of Al compositions in the Fe-Al phase diagram around 25 at.%. The simulated nanocomposites are then quite similar to a single-phase material (Fe${}_{3}$Al with point defects) containing perfect and defected regions.

**Figure 9:**
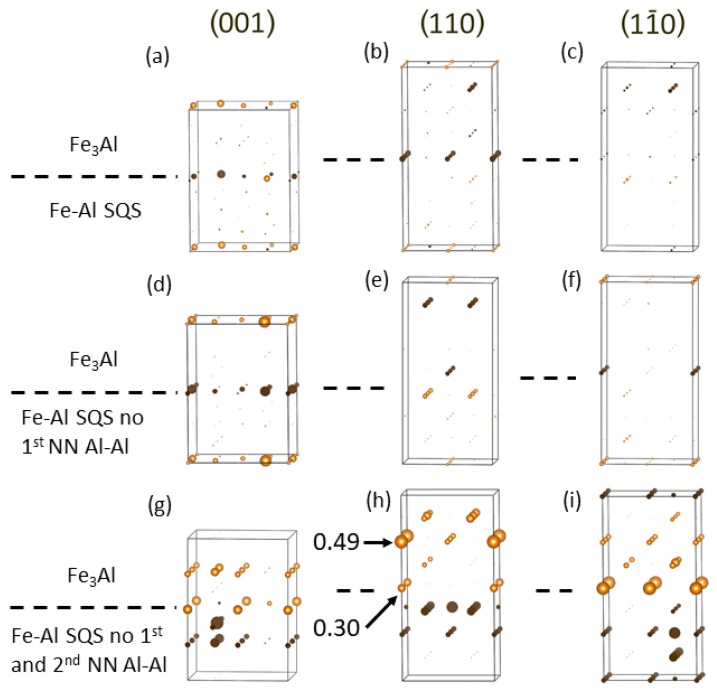
Differences in the magnitude of local magnetic moments of Fe atoms induced by the interfaces within the nanocomposites. They are visualized by the diameter of spheres representing individual Fe atoms (two are listed in subfigure (**h**)). Gold (black) color of the spheres indicate positive (negative) changes. The sub-figures (**a**,**d**,**g**) correspond to the nanocomposites shown in Figure 7a–c, respectively, the sub-figures (**b**,**e**,**h**) to those in Figure 8a–c, respectively, and the sub-figures (**c**,**f**,**i**) corresponds to the nanocomposites with the (1$\bar{1}$0) orientation of the interfaces (not shown). Please note that due to the fact that the differences are rather small, the scaling connecting the value of the difference and the diameter of the spheres is three times bigger than the scaling applied in Figure 2.

Figure 9 shows the differences in the magnitude of local magnetic moments of Fe atoms induced by the interfaces for all three variants of the Fe-Al phase and the three orientations of interfaces.

The changes are indicated by the diameter of spheres representing individual atoms. They are rather small, up to about 0.5 $\mu_{B}$. Let us note that in order to make the spheres representing individual Fe atoms in Figure 9 visible also in the case of very small values of the changes, the scaling connecting (i) the value of the difference on one hand and (ii) the diameter of the sphere in Figure 9 on the other hand, is three times bigger than the scaling applied in Figure 2 above. When inspecting the changes for different orientation of interfaces (rows of subfigures in Figure 9) we find that they depend on the orientation and when analyzing them for different models of the Fe-Al phase (columns of subfigures in Figure 9) we identify that the magnetic moments of Fe atoms sensitively respond to the differences in the distribution of atoms. But the actual changes are clearly rather small.

## 5. Conclusions

We have performed a series of ab initio calculations to examine magnetic and electronic properties of two phases appearing in binary Fe-Al-based nanocomposites. In particular, a disordered solid solution with 18.75 at. % Al in body-centered-cubic (bcc) ferromagnetic (FM) iron, so-called α-phase, was studied together with the ordered intermetallic compound Fe${}_{3}$Al. By comparing results for three different special quasi-random structures (SQS) for the α-phase which differ in the distribution of atoms (they are with or without 1st and/or 2nd nearest-neighbor Al-Al pairs) we found the local magnetic moments of iron atoms clearly affected by the chemical composition of neighboring coordination shells. In particular, the local magnetic moments decrease (increase) with the concentration of Al in the 1st (2nd) coordination shell.

In connection with the Stoner model, a similar tendencies were found in the density of states of individual Fe atoms at the Fermi level as a function of the Al concentration in the 1st and 2nd NN shell. Further, when simulating Fe-Al/Fe${}_{3}$Al nanocomposites (superlattices) changes of local magnetic moments of the Fe atoms (up to 0.5 $\mu_{B}$) are found but they depend sensitively on both the distribution of atoms in the Fe-Al α-phase and the crystallographic orientation of the interfaces. Our findings aim at stimulating further research of coherent nanocomposites of magnetic materials, for example experimental studies employing the Extended X-Ray Absorption Fine Structure (EXAFS) technique and, in particular, the multiple-scattering approach to EXAFS analysis, called GNXAS [77,78], with which it is possible to calculate two-, three- and four-atom correlation functions.

## Appendix A

**Table A1:** Atomic positions (expressed as fractions of supercell dimensions) within the computational supercells shown in Figure 1. Aluminium positions are the first six rows in the case of Fe-Al variants and the first eight rows in the case of Fe${}_{3}$Al.

Fe-Al SQS			Fe-Al SQS no 1NN			Fe-Al SQS no 1&2NN			Fe${}_{3}$Al		
**1**	**1**	$\sqrt{2}$ **/2**	**1**	**1**	$\sqrt{2}$ **/2**	**1**	**1**	$\sqrt{2}$ **/2**	**1**	**1**	$\sqrt{2}$ **/2**
0.75	0.75	0.50	0.50	0.75	0.25	0.25	0.00	0.75	0.25	0.00	0.75
0.25	0.00	0.75	0.75	0.00	0.75	0.25	0.50	0.75	0.75	0.00	0.75
0.50	0.75	0.75	0.25	0.00	0.25	0.00	0.25	0.25	0.25	0.50	0.75
0.00	0.75	0.75	0.25	0.50	0.25	0.00	0.75	0.25	0.75	0.50	0.75
0.25	0.50	0.25	0.50	0.25	0.25	0.50	0.25	0.25	0.00	0.25	0.25
0.50	0.50	0.50	0.50	0.25	0.75	0.50	0.75	0.25	0.00	0.75	0.25
0.50	0.25	0.25	0.75	0.50	0.75	0.75	0.50	0.75	0.50	0.25	0.25
0.75	0.50	0.25	0.00	0.75	0.75	0.75	0.00	0.75	0.50	0.75	0.25
0.00	0.25	0.75	0.50	0.75	0.75	0.00	0.25	0.75	0.00	0.25	0.75
0.00	0.75	0.25	0.00	0.75	0.25	0.00	0.75	0.75	0.00	0.75	0.75
0.00	0.00	0.00	0.00	0.25	0.25	0.50	0.25	0.75	0.50	0.25	0.75
0.00	0.25	0.25	0.75	0.00	0.25	0.50	0.75	0.75	0.50	0.75	0.75
0.25	0.00	0.25	0.25	0.50	0.75	0.25	0.00	0.25	0.25	0.00	0.25
0.50	0.75	0.25	0.75	0.50	0.25	0.75	0.00	0.25	0.75	0.00	0.25
0.75	0.75	0.00	0.25	0.00	0.75	0.25	0.50	0.25	0.25	0.50	0.25
0.25	0.50	0.75	0.00	0.25	0.75	0.75	0.50	0.25	0.75	0.50	0.25
0.75	0.50	0.75	0.75	0.75	0.50	0.00	0.00	0.00	0.00	0.00	0.00
0.00	0.50	0.00	0.50	0.50	0.50	0.50	0.00	0.00	0.50	0.00	0.00
0.50	0.25	0.75	0.00	0.00	0.00	0.00	0.50	0.00	0.00	0.50	0.00
0.75	0.00	0.25	0.75	0.75	0.00	0.50	0.50	0.00	0.50	0.50	0.00
0.25	0.25	0.50	0.00	0.50	0.00	0.00	0.00	0.50	0.00	0.00	0.50
0.50	0.00	0.50	0.25	0.25	0.50	0.50	0.00	0.50	0.50	0.00	0.50
0.00	0.00	0.50	0.50	0.00	0.50	0.00	0.50	0.50	0.00	0.50	0.50
0.50	0.50	0.00	0.00	0.00	0.50	0.50	0.50	0.50	0.50	0.50	0.50
0.25	0.25	0.00	0.50	0.50	0.00	0.25	0.25	0.00	0.25	0.25	0.00
0.75	0.25	0.00	0.25	0.25	0.00	0.75	0.25	0.00	0.75	0.25	0.00
0.25	0.75	0.00	0.75	0.25	0.00	0.25	0.75	0.00	0.25	0.75	0.00
0.75	0.00	0.75	0.25	0.75	0.00	0.75	0.75	0.00	0.75	0.75	0.00
0.50	0.00	0.00	0.50	0.00	0.00	0.25	0.25	0.50	0.25	0.25	0.50
0.75	0.25	0.50	0.75	0.25	0.50	0.75	0.25	0.50	0.75	0.25	0.50
0.25	0.75	0.50	0.25	0.75	0.50	0.25	0.75	0.50	0.25	0.75	0.50
0.00	0.50	0.50	0.00	0.50	0.50	0.75	0.75	0.50	0.75	0.75	0.50
